# Social aspects of childlessness experiences in midlife and late adulthood: a scoping review

**DOI:** 10.1007/s10433-025-00877-7

**Published:** 2025-08-07

**Authors:** Wenqian Xu, Jianmei Zhou, Federica Previtali, Bussarawan Teerawichitchainan

**Affiliations:** 1 Department of Health Sciences, Faculty of Medicine, Lund University, Box 117, 22100 Lund, Sweden; 2 Irish Centre for Social Gerontology, University of Galway, Galway, Ireland; 3 Faculty of Social Sciences, University of Helsinki, Helsinki, Finland; 4 Department of Sociology and Anthropology, National University of Singapore, Singapore, Singapore; 5 Centre for Family and Population Research, National University of Singapore, Singapore, Singapore

**Keywords:** Childlessness, Heterogeneity, Meaning-making, Support systems

## Abstract

Despite its increasing prevalence worldwide, ageing without children remains insufficiently studied. This article presents a scoping review of existing qualitative research examining the social aspects of childlessness in midlife and later adulthood. A total of 3444 papers were retrieved from nine electronic databases (Web of Science, PubMed, CINAHL Complete, APA PsycINFO, SocINDEX, Academic Search Complete, Humanities International Complete, Scopus, and Embase) and supplemented with a grey literature search using Google Scholar. Twenty-five articles were included. Four interconnected themes were found to relate to the experiences of childless adults in midlife and later adulthood: (1) *living within structural contexts* highlights how social norms, life course disadvantages, and policy frameworks affect well-being, access to services, and parenthood possibilities; (2) *navigating care and support* captures the varied ways childless adults access support through informal and formal care systems; (3) *making sense of childlessness* focuses on how individuals interpret their childless status; and (4) *carving out control* describes how individuals adapt to non-parenthood. These findings demonstrate the diversity, complexity, and evolving nature of childlessness experiences among middle-aged and older adults. However, this review also revealed several gaps in the existing literature, highlighting areas for future examination, such as experiences of childlessness among LGBTQ + individuals, how policy frameworks shape lived experiences, and how targeted support interventions may address the needs of childless populations.

**Supplementary Information:**

The online version contains supplementary material available at 10.1007/s10433-025-00877-7.

## Introduction

Childlessness is a multifaceted phenomenon encompassing both women and men, who have never had biological, adoptive, foster, or stepchildren (Albertini and Kohli 2017; Sobotka 2017). With shifting demographic and family patterns, the number and proportion of childless individuals are increasing globally. In Europe, the prevalence of permanent childlessness (i.e. having no children by age 42) among women born during 1900–1972 followed a U-shaped trend (Sobotka 2017). In East Asia, permanent childlessness has also increased sharply, rising from 4 to 12% among women born in the 1950s to as high as 28% in Japan and 26% in Singapore among those born in 1972 (Sobotka 2021). Research further shows that the pathways to childlessness are typically shaped by various life course factors such as poor health (Gray et al. 2013; Heaton et al. 1999), the absence of stable co-residential relationship (Jalovaara and Fasang 2017), and delayed parenthood (Kneale and Joshi 2008). Childlessness also results from a conscious choice, which has increasingly been viewed as a legitimate lifestyle decision (Shapiro 2014). Voluntary childlessness has been linked to broader societal changes, including concerns over children’s quality of life (Helm et al. 2021), and shifts away from traditional notions of parenthood, and the pursuit of alternative sources of life satisfaction such as professional development and meaningful non-parental relationships (Bastianelli 2024; Rojas Betancur et al. 2023).

Existing literature on childlessness has contributed valuable insights into socio-demographic profiles, determinants of childlessness, and implications for well-being across the life course. On one hand, research utilizing large-scale datasets highlights diverse pathways and consequences of childlessness (Agrillo and Nelini 2008; Graham 2015; Sakman 2021; Wang et al. 2024). Evidence suggests that childless individuals form a heterogeneous group, differing in motivations, circumstances, and health outcomes (Poston and Cruz 2016; Quashie et al. 2021; Schroder-Butterfill and Kreager 2005;). This diversity spans never-married individuals, married couples who choose not to have children, and those involuntarily childless due to infertility (Dykstra and Hagestad 2007). On the other hand, qualitative studies examining lived experiences of childless individuals reveal the complex and intersectional ways in which gender, sexuality, partnership status, economic conditions, and societal expectations shape their experiences (Křenková, 2019; Shapiro 2014).

Notably, childlessness carries distinct personal and social implications in midlife and later adulthood, influencing how individuals navigate ageing, identity, social connection, and generativity (i.e. the desire to guide and support the next generation and a key element of midlife development) (Teerawichitchainan and Ha 2024). For many, particularly those who are involuntarily childless, childlessness represents a missed life goal that disrupts expected life trajectories (Hagestad and Call 2007; Hansen et al. 2009). Since parenthood is closely linked to generativity, some childless individuals may experience diminished generative drive in later life (McAdams and Logan 2004). Others, however, may seek alternative forms of generativity, such as caring for relatives mentoring younger colleagues, or taking on quasi-parental roles for their nieces and nephews (Milardo 2005). Many non-parents report positive identities, life satisfaction, and perceived advantages*.* For example, childless adults (especially highly educated women) often enjoy greater personal freedom and increased economic and occupational opportunities, which can lead to greater financial security in later life (Agrillo and Nelini 2008; Gillespie 2003). They tend to cultivate diverse social relationships and contribute through volunteering and community engagement (Albertini and Kohli 2009; Teerawichitchainan et al. 2024). Support networks become increasingly important as one ages. While childless older adults often receive occasional support from extended family, friends, or neighbours, they are more likely to rely on formal care services (Deindl and Brandt 2017) and lack critical forms of intensive or personal care during periods of poor health (Albertini and Mencarini 2014; Wenger 2009).

In this study, we define midlife and late adulthood as beginning around age 49, a point marking the sharp decline in reproductive potential. For women, this aligns with the end of reproductive capability (World Health Organization 2020), while for men, fertility outcomes significantly worsen after this age (Horta et al. 2019). Our focus is on the social aspects of childlessness—how childless individuals experience, negotiate, and are shaped by their social environments during these life stages. In doing so, we highlight the ways individuals construct their own narratives, identities, and sources of fulfilment in response to or in tension with prevailing societal expectations (Fieldsend and Smith 2020; Shapiro 2014). These experiences are shaped by gender, social class, sexuality, and cultural contexts. For instance, in India, involuntarily childless women often face stigma across personal, familial, and healthcare domains, reflecting the deep cultural associations between motherhood and feminine identity (Mehta and Kapadia 2008). By contrast, a cross-national study in 25 European countries found that voluntary childlessness is more strongly disapproved of in men than women, particularly in more gender-equal societies (Rijken and Merz 2014). In the UK, childfree women from higher social classes have been found to resist feminine norms tied to motherhood (Gillespie 2003). Similarly, childless LGBTQ + individuals challenge heteronormative expectations by embracing “families of choice” and “co-independence” in their intimate lives (Clarke et al. 2018).

This study’s focus on the social aspects of childlessness holds significant implications for practice and policy. First, it challenges deficit-based narratives that portray childless middle-aged and older individuals as lonely, impoverished, or overly dependent on social and health services (Cwikel et al. 2006). These stereotypes often rooted in sociocultural expectations around family and legacy contribute to persistent stigma, particularly for women, whether childlessness is voluntary or involuntary (Miall 1986; Shapiro 2014). Second, by highlighting the complexity and diversity of childlessness experiences, the study advocates for an intersectional perspective that considers how gender, class, sexuality, and other social identities shape individual agency and life choices within broader social-structural contexts. These insights can inform the development of more responsive policies and interventions that addresses the unique needs of childless individuals across the life course.

This scoping review synthesises existing research on the social dimensions of childlessness in midlife and late adulthood and identifies key gaps in the literature. By focusing exclusively on qualitative studies, the review provides a nuanced and in-depth understanding of lived experiences of childless individuals. The findings aim to inform policy development and support societal adaptation in response to the unprecedented global rise in childlessness.

## Methods

This scoping review was conducted using a five-step methodological framework (Arksey and O’Malley 2005; Levac et al. 2010), consisting of: (1) identifying the research questions, (2) search strategy, (3) study selection, (4) charting the data, and (5) collating, summarising, and reporting the results. We searched multiple databases of indexed literature, including general, multidisciplinary, medical, as well as social science databases, based on childlessness-related concepts. Inclusion and exclusion criteria were applied iteratively. The reporting of the scoping review was guided by the Preferred Reporting Items for Systematic Reviews and Meta-Analyses (PRISMA) checklist (Page et al. 2021).

### Step 1: identified research questions

The initial step involved formulating the research questions, which was achieved through an online meeting between the authors. Research questions were drawn from previous literature and include: What are the social aspects of the experiences of childlessness in midlife and late adulthood? What key insights does the existing literature provide about these social aspects?

### Step 2: search strategy

Search terms were identified through a review of relevant literature, pilot searches, and consultation with an information specialist at Lund University, followed by discussion within the research team. Our search strategy follows the PCC (population, concept, context) framework to ensure comprehensive coverage of the relevant literature. “Population” refers to childless individuals, defined as those who have never had children, excluding bereaved parents who have lost their only child. While terms such as “involuntary childlessness,” “childless-by-choice,” and “childless-by-circumstance” were not explicitly included in the initial search, they are often encompassed within the broader term “childless.” We excluded terms like “without child/kin” and “no child/kin” from the final set of keywords due to low match rates. Keywords related to “midlife and late adulthood” were added at a later stage rather than in the initial search to ensure that no relevant studies were overlooked, as such terms are often used inconsistently or only mentioned in the full text. “Concept” focuses on the social aspects of lived experiences. The review includes the keyword “(dis-)satisfaction” to capture people’s views on their lives, surroundings, or the services they use. Additionally, we excluded studies (see Table 2) focused on medical, clinical, or therapeutic interventions. This approach enables our review to capture the broadest possible range of social experiences related to childlessness. “Context” did not have specific search terms applied. See Table 1 for the complete list of search terms.

**Table 1:** Search terms

PCC component	Search terms
Population	(childless* OR childfree OR kinless*)
Concept	(experience* OR lived experience OR satisfaction OR dissatisfaction) AND NOT (medical OR clinical OR therap*)
Context	None

Combinations of the terms listed in Table 1 were searched across nine electronic databases, targeting abstracts or keywords. These included Web of Science, PubMed, Scopus, Embase, CINAHL Complete (EBSCOhost), APA PsycINFO (EBSCOhost), SocINDEX (EBSCOhost), Academic Search Complete (EBSCOhost), and Humanities International Complete (EBSCOhost). This search yielded 3414 studies. After removing 1996 duplicates, 1418 studies remained. Grey literature was searched via Google Scholar, and the reference lists of all included articles were screened for additional relevant studies. The last two authors contributed to refining the search methodology by reviewing database selection, improving search terms, and conducting initial searches. All sources were collected and organised using Covidence, and detailed records were retained to ensure the replicability of the strategy.

### Step 3: study selection

The study selection followed a two-stage screening process based on predefined inclusion and exclusion criteria (Table 2). We excluded mixed methods studies, as many did not sufficiently report qualitative components (e.g. participant demographics) or failed to clearly distinguish qualitative from quantitative findings, making consistent data extraction unfeasible. In the first stage, two authors individually screened the titles and abstracts of all retrieved records (*n* = 1418) for relevance. Studies identified as potentially eligible (*n* = 434) proceeded to full-text screening. A total of 30 records were excluded due to the unavailability of full texts, despite extensive efforts to retrieve them, including contacting corresponding authors and searching academic repositories such as ResearchGate, Academia.edu, and publisher websites. The remaining articles (*n* = 405) were assessed in full by the same two authors, independently against the inclusion and exclusion criteria, resulting in 16 eligible studies. Discrepancies between reviewers were resolved through discussion. To supplement the database search, the reference lists of included studies were reviewed, and the top 200 results from Google Scholar were screened using the original search terms, yielding an additional nine studies. As few further relevant articles were identified after reviewing subsequent pages of Google Scholar results, this suggested that the search had likely identified as many relevant studies as possible within the review’s scope.

**Table 2:** Inclusion and exclusion criteria

Criteria	Inclusion	Exclusion
Language	English	All other languages
Publication year	Published up to 31 December 2024	Published on or after 1 January 2025
Types of studies	Empirical qualitative studies	Letters, comments, notes, editorials, meeting abstracts, theses, monographs, reviews, and quantitative or mixed-method studies
Phenomenon of interest	Social aspects of childlessness experience (interpersonal, structural, and cultural dimensions through which individuals understand, navigate, and are shaped by their childless status across various life domains)	No focus on childlessness; studies focused on medical, clinical, or therapeutic interventions (e.g. infertility treatment, assisted reproductive technology, or egg freezing procedures)
Study participants	Individuals (women and men) aged 49 and above, described using terms such as “middle-aged,” “midlife,” “later life,” “late adulthood,” “older adults,” or “old age”	Participants under 49 years old

### Step 4: data extraction

The first two authors extracted data from the included 25 articles using a bespoke data extraction tool designed by the research team (see Table 3) comprising objectives, location, methods, sample size, participant demographics, and key findings.

**Table 3:** Overview of eligible studies

Author(s)	Study objective	Study location	Methods	Sample size	Childless participant characteristics	Main findings
Abramowska-Kmon et al. (2023)	Explore the challenges faced by older adults who do not have any children, their strategies to address these challenges, and the contributing factors to subjective well-being	Poland (rural and urban)	In-depth interviews; qualitative content analysis	42 participants	Aged 65+ 20 men and 22 women 10 married, 22 single and 10 widowed	Participants encountered significant challenges related to loneliness, isolation, insufficient practical support and care in case of dependency, and dying childless. They adopted coping strategies throughout their lives, such as promoting healthy ageing and assisting older individuals in expanding their social networks. Study participants’ challenges and coping strategies evolved over time, reflecting changes in their biographies. Their actions were strongly influenced by their personalities and lifestyles in their younger years
Alexander et al. (1992)	Explore the experience of regrets about childlessness in older women	USA	Interviews; qualitative analysis	90 participants	Aged 60 + All women 30 never married, 29 married and 31 widowed	Regrets among childless women are shaped and formed in the context of a culture that predominantly defines womanhood through childbearing and forces women to evaluate themselves against the pressure of this cultural prescription. This influence is evident in their social interactions and personal assessments of lives deviating from the normative path, which can create and intensify feelings of regret about childlessness
Allen and Wiles (2013a)	Explore how older people describe their paths to late-life childlessness	New Zealand, Auckland	Semi-structured interviews; analysis of narratives	38 participants	Aged 63–93 29 women and 9 men 27 European New Zealanders, 1 Māori and 10 others 19 single, 9 widowed, 6 married, 4 divorced and 1 separated	Childlessness is a fluid identity within diverse narratives across time and circumstances. It, for some, was an active choice to break a cycle of family violence; for others, it resulted from social upheaval. Over time, it elicited a mix of grief and relief, viewed either as a discernment in refusing parenthood at any cost or as something that felt natural within a meaningful life
Allen and Wiles (2013b)	Encourage gerontologists to apply positioning theory in ageing research, using examples from research with childless older adults	New Zealand, Auckland	Interviews; positioning analysis	38 participants	Aged 63–93 Women and men Single, married, divorced and separated Non-heterosexual (some)	Participants gave accounts of childlessness through complex and shifting positions across storylines. They also expressed shifting and contradictory positions and preferences in relation to residential care. Emotional support was often framed as within the self, rather than in relationship with others
Allen and Wiles (2014)	Provide qualitative understanding of childless older people’s talk about support	New Zealand, Auckland	Semi-structured interviews; analysis of narratives	38 participants	Aged 63–93 29 women and 9 men 27 European New Zealanders, 1 Māori and 10 others 19 single, 9 widowed, 6 married, 4 divorced and 1 separated	Participants understood support in varied ways. It was accepted when linked to illness, reciprocity, or a trusted giver, but resisted when tied to loss of independence or assumptions of incapacity. “Oldness” was often seen negatively—both as a reason for needing support and a consequence of receiving it
Black and Hannum (2015)	Explore the experiences of childless older women in relation to ageing, time, spirituality, and future care needs	USA, mid-Atlantic states	One-on-one interviews once a week for three consecutive weeks; phenomenological data analysis	4 participants	Born between 1916 and 1945 All women Never-married	Ageing, time remaining, and spirituality were core concepts in participants' reflections concerning the planning of future care needs through interdependence. Their plans were based on unknowingness and limited familial resources in regard to confronting chronic or acute health challenges. Their personal spiritual experiences were expressed through a growing desire for connection with God and others
Boker Gonen et al. (2024)	Explore the experiences of older childless women	Israel	Semi-structured interviews; thematic analysis	20 participants	Aged 61–95 All women 14 single, 2 married, 3 widowed and 1 divorced Heterosexual	Participants described the circumstances that led to their childlessness, expressing feelings ranging from regret to reconciliation in later life, along with criticism of parenting. They emphasised a strong desire for freedom and the personal resources invested to achieve it, despite social and personal challenges. Their narratives also reflected contrasting views—seeing life as both shaped by fate and as something they could actively control
Chen and Lou (2023)	Explore the role of the next of kin in the residential decision-making of childless older adults	China, southwest area (rural)	Semi-structured interviews, grounded theory data analysis	27 participants	Aged 60–92 19 men and 8 women 3 married, 9 separated /divorced/widowed and 15 never-married	Participants tended to choose to age in place if they had a continuous, reliable, and trust relation with next of kin. They co-construct the reasoning behind their residential decisions through negotiations with their kin, involving exchanges for care, maintaining relational intimacy, and relying on filial obligations
Chen and Lou (2023)	Explore how childless older adults in rural China choose between ageing in place and institutionalisation	China, Yunnan province (rural)	Semi-structured interviews, grounded theory data analysis	25 participants	Aged 60–83 18 men and 7 women 15 never-married and 10 married	Participants used the term ku (“bitterness,” metaphorically meaning farming activities) to describe their residential choices. Those able to endure ku, sustaining themselves through farming, preferred ageing in place; others relocated to institutions. They adapted by positively reappraising ku, incorporating it into routines, and transforming it into a toughness identity
de Medeiros and Rubinstein (2018)	Explore how never-married women who do not have biological children identify themselves in terms of age	USA, mid-Atlantic	Semi-structured and open-ended, life-story interviews over three consecutive weeks; thematic analysis	53 participants	Aged 60 + All women 22 African American and 31 White Never married	For women who have never-married and childless, advancing age can be a source of great power and ability if there can be a shift away from how old age “looks” to how old age “feels”. It was contrast between what they should feel and how they viewed themselves
Ebimgbo et al. (2023)	Examine the adequacy of material support systems for childless older adults in southeast Nigeria	Nigeria, Nnewi (urban)	Semi-structured interviews, thematic analysis	12 participants	Aged 75–88 6 women and 6 men 5 married, 5 widowed and 2 single	Participants faced poor economic conditions, receiving irregular and insufficient material support from family, community, and churches. The government offered no provisions
Hadley (2018)	Explore the impacts of involuntarily childlessness on the lives of older men	UK (incl. one participant residing in Thailand)	Semi-structured biographic-narrative interviews	14 participants	Aged 49–82 All men 13 White British and 1 Anglo-Celtic Australian 9 partnered, 7 single and 2 widowed 12 heterosexual and 2 non-heterosexual	For heterosexual men, fatherhood was expected and framed by their cultural, economic, geographic, social, temporal, and view-of-others and view-of-self contexts. Participants’ responses to involuntary childlessness fell into three broad types: aspirational, uncertain, and mediated. In the mediated group, heterosexual men linked acceptance to ageing and intimate relationships, while gay men reflected on how shifts in reproductive technology, family norms, and policy shaped their parenthood possibilities. While some came to terms with childlessness, others continued seeking parenthood or grandparenthood
Hadley (2021a)	Explore the lived experiences of childless men aged 50 to 70 years and who currently or in the past, wanted to be a father	UK (rural and urban)	Auto/biographical qualitative interviews; thematic analysis	14 participants	Aged 49–82 All men 13 White British and 1 Anglo-Celtic Australian 9 partnered, 7 single and 2 widowed 12 heterosexual and 2 non-heterosexual	Childlessness intersects with individual agency, relationships, and sociocultural structures. Participants’ social networks were influenced by factors like familial structure, relationship quality, location, and employment over the life course. Those without siblings depended on distant relatives or fictive kin, while those with siblings relied on relationship quality. Many noted the impact of grandparenthood. LGBT people faced inequalities in social and cultural resources, affecting their social networks
Hadley (2021b)	Examine older men’s experience of involuntary childlessness	UK (rural and urban)	Auto/biographical qualitative interviews; thematic analysis	14 participants	Aged 49–82 All men 13 White British and 1 Anglo-Celtic Australian 9 partnered, 7 single and 2 widowed 12 heterosexual and 2 non-heterosexual	Participants voiced concerns about ageing, specifically the potential decline in physical and mental health. For those without a partner or designated power of attorney, additional anxieties included limited relationships, social isolation, loneliness, and end-of-life issues. Participants also expressed concerns about how solo-living older individuals would access health and care services. Those caring for older relatives often contemplated the potential lack of reciprocal care in their own old age, a concern amplified for those without siblings. The term “hypochondriac” was used by participants as they navigated dominant sociocultural “virility” discourses surrounding men and masculinity in their utilisation of health services. Non-heterosexual men expressed concerns about potential discrimination when accessing residential care in later life. The desire to maintain identity and autonomy was reflected in a preference for residential care over burdening close family members. Embracing an aged identity often led to a sense of liberation, freeing individuals from conforming to social hierarchies and potentially accepting childlessness
Ikels (1988)	Explore the role of siblings in the support networks of the never-married and/or childless older adults	USA, Greater Boston	Interviews; qualitative case analysis	34 participants	Aged 50 + Women and men Irish American 15 never-married and 19 partnered	Physical presence and proximity of siblings, along with cultural and familial influences shaping their expectations of each other, are relevant to the support networks of childless older adults. In particular, sister-sister households living arrangements prevail, followed by sister-brother households, and lastly, brother-brother households. Sisters commonly reside in close proximity, while brothers tend to be more dispersed, reflecting expectations linked to settlement patterns for newly married couples
Johnson and Catalano (1981)	Describe the situation of childless older adults confronted by a dependency crisis when their health deteriorated to a point requiring hospitalisation	USA, San Francisco	Interviews; comparative analysis and content analysis	28 participants	Mean age 74.5 years Women and men White 10 married and 18 unmarried	The childless married were more isolated and tended to rely primarily upon each other, while the unmarried were more resourceful in using a long-term accumulation of social resources to meet their needs. Where no spouse was available, distant relatives provided childless older adults with perfunctory care such as arranging for hired help, offering legal and financial advice, or giving occasional transportation. Marital status affects the adaptation to childlessness. The childless married experienced a progressive social regression or withdrawal from other social ties into the interdependence of the marital relationship. The childless unmarried prepared for potential dependency by building a network of resources within their extended family and among friends in preparation for the possibility of dependency
Lamb (2001)	Explore a life story of a childless widow	India, West Bengal	Life story interview; narrative analysis	1 participant	Late fifties or early sixties A woman Brahman Widowed at a young age	The participant saw widowhood as a core part of her identity. She followed the widow’s code to express lasting devotion to her husband and to maintain her identity as a wife. She also believed that having children would have eased her suffering, linking childlessness to feelings of absence and longing
Ng et al. (2020)	Examine how lifelong advantages and disadvantages affect housing stability for childless, low-income older adults in their later years	Malaysia, Kuala Lumpur	Semi-structured interviews; thematic analysis	34 participants	Aged 62–82 24 women and 10 men 19 Chinese, 10 Malay, 4 Indian and 1 other 2 partnered and 23 single and 9 widowed	Participants’ ability to secure housing in old age was affected by various factors, such as family poverty, limited job opportunities, and poor health. Their individual decisions, social history, and background impacted access to housing resources over their life course and influenced housing arrangements in old age. Policy factors, for example, eligibility criteria for public housing and retirement entitlement initiatives, also failed to address inequality in housing in old age
Rubinstein et al. (1991)	Explore the central, compelling, enduring, or significant relationships of never married, childless older women throughout their lifetime	USA	Interviews; qualitative analysis	31 participants	Aged 60 + All women Never married	Participants navigate three relationship types: blood ties, friendships, and “constructed” kin-like nonkin relations. Actively overcoming cultural constraints, they employ two key strategies for the expectation of care in later life: strengthening ties through shared substance, manipulating conduct codes in collateral ties; and creating shared identity in substance-lacking relationships, utilising conduct codes despite challenges. The clarity of the “code for conduct” is evident in lineal relationships, but ambiguity persists in non-primary blood relatives and constructed relationships, requiring a complex negotiation process
Silva et al. (2024)	Explore the transmission of material inheritances by older childless adults	Portugal	Semi-structured interviews; content analysis	13 participants	Aged 65 + 12 women and 1 men 6 single, 2 married or living with partner, 1 divorced and 4 widowed	Childless donors tend to get more legal advice, distribute more in life, and define in life how they want the distribution to occur (by making a will and informing potential heirs). Childless donors use as guiding values mainly strategic exchange and reciprocity. Childless donors seek to prevent family conflicts, both before and after death, with outcomes often marked by either separation and discord or unity among potential heirs
Stahnke et al. (2020)	Gain a deeper understanding of the overall life satisfaction of older adult women who have not had children	USA, Southeast Florida	Open-ended interviews; interpretative phenomenological analysis	14 participants	Aged 67–87 All women White/Caucasian 4 never-married and 9 ever-married	Participants reported an increase in life satisfaction as they aged, attributing it to accepting unfulfilled goals and embracing the positives of being childfree. Close relationships, with both friends and partners, were identified as crucial for enhancing life satisfaction. Participants navigated, tolerated, or persisted through various stressors and obstacles, enabling them to thrive and achieve life satisfaction
Stahnke et al. (2022)	Understand the life satisfaction experienced by childfree women over the age of 65, considering some assumption in society that parenthood is the best path to fulfilment	USA, South Florida	Survey and semi-structured interviews; thematic analysis and descriptive analysis	14 participants	Aged 67–87 All women White/Caucasian 4 never-married and 9 ever-married	Participants conveyed a sense of life satisfaction and ego integrity, with the majority adapting and embracing their childfree lives, finding fulfilment. Many also experienced a sense of freedom. Participants faced pressure and stigma because of their childfree status
Stahnke et al. (2024)	Explore the dissatisfied experiences of a childfree older woman	USA, Southeast	Interview and survey; narrative analysis with a thematic approach	1 participant	Aged 73 A woman White Heterosexual	The participant linked her childlessness to a dysfunctional family background and fear of starting her own family. She regretted missed opportunities, felt she hadn’t lived with integrity, and lacked life satisfaction despite some connection with younger generations
Stegen et al. (2021)	Investigate the reasons for and experiences of voluntary childlessness throughout the life course	Belgium, Flanders and Brussels	Life-story interview; thematic analysis	13 participants	Aged 61–84 7 women and 6 men 3 married, 6 single and 4 widowed	Regarding reasons for voluntary childlessness, four profiles emerge: the liberated careerist prioritising work over childcare, the social critic examining society, the acquiescent partner attributing the choice to their spouse, and a broader profile encompassing diverse life course circumstances leading to voluntary childlessness. Participants felt various emotions, from acceptance to regret, relief, and loss, including missing familiarity with trends, assistance, and children's company. Some participants, lacking children for support, were worried about increased care needs. Several had already arranged for physician-assisted death in case of urgent care requirements
Wenger (2001)	Identify the pathways and adaptations to childlessness	UK, Wales	Survey, structured interviews and observation; longitudinal analysis	65 participants	Aged 65 + 30 women and 25 men 32 never-married and 33 ever-married	Women generally show more positive adaptations to childlessness than men. Never-married women are more independent and outgoing, often working in socially interactive jobs. Many childless married women, after marriage, had close relationships with their husbands and adopted an independent, self-sufficient lifestyle in widowhood

### Step 5: data analysis, and synthesis

We conducted a thematic analysis of the main findings from eligible studies, following Braun and Clarke’s (2006) approach and the scoping review protocol by Levac et al. (2010). Adopting an inductive approach, the first two authors collaboratively conducted open coding of the extracted findings using Atlas.ti, focusing on meanings, experiences, and social contexts. The codes were then compared and refined to capture underlying conceptual ideas. Related codes were subsequently grouped into clusters reflecting broader conceptual patterns across studies. Through iterative discussion, four themes were consolidated to illustrate how childlessness is experienced, negotiated, and understood in relation to structural conditions and individual agency.

## Results

**Fig. 1:**
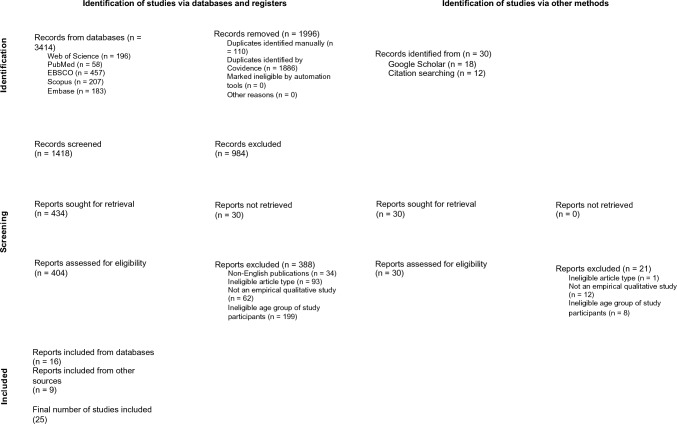
PRISMA flowchart (scoping reviews [ScR]) applied to the current study

Of the 1418 records retrieved through database searches, a total of 16 studies met the inclusion criteria. An additional nine studies were identified through backward reference checking and targeted screening of Google Scholar records. In total, 25 studies were included (Fig. 1).

### Overview of the included studies

The included studies were developed in the USA (*n* = 9; 36%), UK (*n* = 4; 16%), and New Zealand (*n* = 3; 12%). 80% of the studies are based in high-income countries (Table 3). Nine studies focused exclusively on women, while three specifically addressed men. Data collection in all the studies involved interviews. Qualitative data analysis included content, narrative, thematic, and interpretive phenomenological analyses.

### Social aspects of childlessness experiences in midlife and late adulthood

Our analysis identified four interconnected themes (Table 4).

**Table 4:** Themes and subthemes

Themes	Subthemes
Living within structural contexts	Dealing with social norms about having children Navigating policy frameworks Linking later-life well-being to life course dynamics
Navigating support systems in response to care needs	Accessing and providing care through kinship Negotiating institutional and social care
Making sense of childlessness	Experiencing mixed and complex feelings Giving accounts for childlessness Constructing self-identity without parenthood
Carving out control through adaptation and planning	Adapting to childlessness Planning for the future

### Living within structural contexts

The findings are organised into three subthemes, each highlighting structural forces encountered by childless adults. (a) *Dealing with social norms about having children*: Stigma and stereotypes related to childlessness were evident across diverse cultural contexts and among various childless older adult groups. Across contexts such as the USA, Israel, and the UK, pronatalist and heteronormative norms contributed to both interpersonal and internalised stigma, shaping how individuals made sense of their lives and reproductive choices (Alexander et al. 1992; Boker Gonen et al. 2024; Hadley 2018, 2021b; Stahnke et al. 2022). This was evident among voluntarily or involuntarily, heterosexual or non-heterosexual childless individuals.

(b) *Navigating policy frameworks*: Policies that prioritise individuals with family ties or marital histories are experienced as limiting and discriminatory by never-married individuals, failing to account for the diverse circumstances and support needs within the population. For instance, childless older adults in Malaysia experienced exclusion from public housing and retirement benefits due to family-centred eligibility criteria, highlighting structural barriers to housing (Ng et al. 2020). In the UK, prior to the Equality Act (2010), non-heterosexual men faced structural barriers to adoption, and by the time legal opportunities expanded, age norms constrained their opportunities to pursue parenthood (Hadley 2018).

(c) *Linking later-life well-being to life course dynamics*: Evidence suggests that childless older adults’ living conditions and well-being are shaped by early life events and experiences. For instance, adverse life course events (e.g. family poverty, poor health, and job loss) limited the ability of Malaysian childless older adults to accumulate financial resources and constrained their housing options (Ng et al. 2020). However, childless older men in the UK maintained resilient networks formed through sibling ties, past employment, and leisure activities (Hadley 2021a). In Poland, those socially active in earlier life were more likely to sustain support networks in old age, suggesting continuity over the life course (Abramowska-Kmon et al. 2023).

### Navigating support systems in response to care needs

The findings comprise two subthemes. (a) *accessing and providing care through kinship*: Their experiences of receiving care are shaped by specific conditions and complex considerations. In New Zealand, childless older adults were more likely to accept support when it was linked to illness, reciprocity, or a trusted care provider, but tended to resist support when it implied incapacity or undermined their independence (Allen and Wiles 2014). Familial sources of care vary depending on relationship quality and proximity, with partners and siblings often playing a significant role when available. In the USA, married childless older adults relied primarily on their spouses during health-related dependency crises, while those without spouses turned to other relatives for limited practical assistance, such as hiring help or providing legal and financial guidance (Johnson and Catalano 1981). Sister–sister households among Irish Americans provided support when siblings lived nearby (Ikels 1988). In the UK, childless older men without siblings frequently turned to distant relatives or fictive kin to meet their informal care needs (Hadley 2021a). While often depicted as care recipients, childless older adults also engaged in supporting ageing parents, siblings, or younger generations (Hadley 2021b).

(b) *Negotiating institutional and social care*: Across varied settings, childless older adults may engage with wider social organisations for assistance, including the state welfare, community organisations, religious institutions, and self-help groups, though these supports are reported to be inadequate in resource-constrained settings (e.g. Chen and Lou 2023; Ebimgbo et al. 2023; Ng et al. 2020). In New Zealand, childless adults expressed diverse and sometimes contradictory views on residential care, such as a desirable setting if friends were present, and a compromise for specific living arrangements (Allen and Wiles 2013b). In the UK, non-heterosexual childless older men were concerned about discrimination in residential care but often preferred such arrangements to avoid burdening family (Hadley 2021b). In rural China, residential decisions were negotiated with kin, with ageing in place preferred when relationships were reliable and trust-based; otherwise, residential care served as an alternative (Chen and Lou 2023). Such decisions reflect complex negotiations of dignity, autonomy, and available alternatives.

### Making sense of childlessness

The included studies show that the meanings attached to childlessness are shaped through emotional experiences, contextualised life narratives, and intersecting social identities. These insights comprise three subthemes. (a) *Experiencing mixed and complex feelings*: Emotions ranged from freedom to regret, often shaped by whether childlessness was voluntary or involuntary, and by early life contexts. In the USA, voluntarily childfree older women expressed a sense of freedom, ego integrity, and life satisfaction (Stahnke et al. 2022), while those with dysfunctional family background expressed lasting regret, dissatisfaction, and a diminished sense of life integrity (Stahnke et al. 2024). In Belgium, emotional responses were dynamic, from relief and acceptance to subtle grief and regret (Stegen et al. 2021). In New Zealand, Allen and Wiles (2013b) identified childlessness as a fluid identity negotiated over time, framing it either as a purposeful decision or an outcome that aligned with a meaningful life trajectory. Regarding involuntary childlessness, Hadley (2018) identified disenfranchised grief among older men in the UK, often accompanied by feelings of social alienation and marginalisation in relation to social clocks. These findings suggest that emotional responses evolve over time and are shaped by social norms and personal histories.

(b) *Giving accounts for childlessness*: Participants constructed retrospective stories to account for childlessness. Voluntary accounts include those who sought to break cycles of family violence (Allen and Wiles 2013a), those who prioritised career and independence (Stahnke et al. 2020; Stegen et al. 2021), and those who expressed reluctance to have children in a world marked by climate crisis, overpopulation, and political instability (Stegen et al. 2021). In contrast, other accounts emphasised historical and biographical circumstances. Childless older women in Israel cited difficulties finding a suitable partner, being in relationships deemed unfit for parenting, unsuccessful conception attempts, and a conscious decision to avoid single motherhood due to perceived emotional, physical, and social limitations (Boker Gonen et al. 2024). Childless older men in the UK attributed to the absence of a willing partner, perceptions of being too old to parent, infertility or failed reproductive treatments, financial insecurity, life events such as bereavement or missed timing, and limited legal and social pathways to parenthood (particularly for non-heterosexual men) (Hadley 2018).

(c) *Constructing self-identity with or without parenthood:* Childlessness intersects with widowhood and ageing, shaping identity construction. Based on the narrative of a childless widow in India, Lamb (2001) found that the woman upheld her identity through the “widow’s code” and believed that having children would have eased her suffering and the social difficulties faced by widows in Bengali society. In the USA, de Medeiros and Rubinstein (2018) found that never-married, childless women understood ageing in terms of agency, continued engagement, and self-perception, and rejected parenthood as a defining feature of age identity.

### Carving out control through adaptation and planning

Two subthemes emerged. (a) *Adapting to childlessness*: Both voluntarily and involuntarily childless adults adapted by cultivating meaningful relationships and constructing generative roles. Studies from the USA found that childfree women led generative lives in personal and professional contexts through maintaining meaningful social relations and mentoring younger generations through surrogate roles (Rubinstein et al. 1991; Stahnke et al. 2020). Involuntarily childless men in the UK who had experienced unsuccessful in vitro fertilisation or other limiting circumstances came to accept their childlessness by forming non-biological “fictive grandfather” roles through social relationships and family ties (Hadley 2018).

(b) *Planning for the future*: Childless adults proactively prepared for future unknowingness (e.g. anticipated care needs, end-of-life planning, and post-mortem responsibilities). In Poland, childless older adults adopted active ageing strategies to maintain independence and self-reliance (Abramowska-Kmon et al. 2023). Other measures related to improving social and mental well-being were also noted around studies, such as expanding social interactions and networks, and sustaining contacts with friends and extended family as potential sources of informal support (Abramowska-Kmon et al. 2023; Johnson and Catalano 1981; Rubinstein et al. 1991). In Belgium, some childfree older adults arranged for physician-assisted death as a way to manage future care in the absence of close caregivers (Stegen et al. 2021). In Portugal, childless older adults sought to prevent family conflict over material inheritance by seeking legal advice, distributing assets during their lifetime, and clearly articulating their wishes through wills and direct communication with potential heirs (Silva et al. 2024).

## Discussion

Our scoping review mapped existing research on the lived experiences of childlessness in midlife and late adulthood, with a focus on social dimensions. Four interconnected thematic findings were synthesised and presented.

First, we identified several structural forces that shape the lived experiences of childlessness, with sociocultural norms around parenthood emerging as an influential factor across study contexts. Pronatalist norms were evident not only through interpersonal conversations and social expectations but also through the internalisation of these norms by childless individuals. As Lee (2024) argues, pronatalism is neither a universal nor self-evident value, but rather one rationalised by the uncritical adoption of conventional gender norms and normative beliefs that privilege biological parenthood over other life choices and values. Specifically, gender norms remain closely tied to reproductive roles, with femininity associated with motherhood and caregiving, and masculinity linked to fatherhood and social lineage in various cultural contexts (Mehta and Kapadia 2008; Rijken and Merz 2014). Our findings indicate that such norms contribute to complex emotional responses among heterosexual women and men who diverge from them through childlessness (e.g. feelings of othering, perceived loss of social roles, and identity disruption), often prompting a life course process of adaptation in response to social pressure and stigma (Allen and Wiles 2013b; Boker Gonen et al. 2024; Stahnke et al. 2024). Notably, although underrepresented in the literature, the reviewed studies highlight how pronatalist and heteronormative norms distinctly shape childlessness experiences of non-heterosexual individuals. For example, Hadley (2018, 2021a, b) found that some self-identified gay men in the UK internalised the belief that parenthood was only legitimate within heterosexual marriage, while others refrain from engaging in informal grandparent-like roles for fear of being perceived as paedophiles.

Moreover, our findings indicate that age norms significantly shape how childless individuals experience and interpret their childlessness, often in varied and sometimes contrasting ways. These norms are embedded in culturally sanctioned expectations surrounding the appropriate timing of life course events such as marriage and parenthood, and in idealised life course trajectories that prioritise a linear sequence of education, employment, marriage, and childbearing (Elder 1998; Neugarten 1968). One reviewed study found that some childless older women experienced intensified regret as they aged, linked to feelings of missed opportunities, concerns about future care needs, and anxieties regarding generational continuity (Alexander et al. 1992). Such responses may reflect the increasing salience of age-normative pressures, which become more pronounced in later life, especially as individuals confront the irreversible nature of reproductive decisions. However, more recent studies revealed that some childless older adults rejected deficit-based narratives and instead constructed a positive age identity by drawing on experiences of liberation, fulfilment, and accrued wisdom (Black and Hannum 2015; de Medeiros and Rubinstein 2018; Hadley 2021b). These findings suggest that individuals negotiate age-related expectations in diverse ways—affirming, resisting, or reinterpreting age norms.

Another key theme concerns how childless adults navigate care and support in the context of health-related concerns and needs. Several qualitative insights align with quantitative research showing that while childless older adults often rely on a mix of familial and social care resources, those living in settings with limited welfare infrastructure face a heightened risk of inadequate access in formal and intensive support (Deindl and Brandt 2017). However, our review offers a more nuanced perspective: childless adults articulated diverse personal preferences and expectations regarding the types of support they were willing to receive, the circumstances under which they would accept or resist care, and the timing they deemed appropriate. These findings highlight the importance of self-care practices, including proactive planning for future care needs and negotiating care arrangements with significant others.

This scoping review identifies several evidence gaps that warrant further research. First, there is a need to explore the lived experiences of underrepresented childless groups, such as LGBTQ + individuals and childless men, whose perspectives remain largely absent from the existing literature. Future research should also examine how social programmes and policy interventions address the needs and preferences of childless individuals in midlife and late adulthood. Moreover, empirical studies from non-Western countries remain limited; only 5 out of the 25 studies reviewed were based outside Western settings. This restricts the global and cultural scope of current knowledge. Expanding research in underrepresented settings would foster a more inclusive and culturally nuanced understanding of childlessness. Additionally, the impact of socio-economic disadvantages on childlessness experiences remains underexplored, particularly for individuals with low income, rural residents, and those occupying marginalised social positions such as ethnic minorities, migrants, and refugees. Addressing these gaps is essential for informing equitable policies and practices that promote the health and well-being of diverse childless populations across the life course.

Our review highlights the need for policy and practice to consider the lived experiences of childless individuals in midlife and later adulthood. Reviewed studies from the UK, Malaysia, and Nigeria highlighted how existing policies often restricted childless individuals’ access to essential services such as public housing, retirement benefits, and care provision, and constrained legal rights related to adoption (Ebimgbo et al. 2023; Hadley 2018; Ng et al. 2020). Policies that presume older adults will be cared for by adult children or spouses should be revised to ensure equitable access to personal care services, independent living such as subsidised home modifications and transportation, and income security measures that promote autonomy. Care interventions should be culturally sensitive and attuned to the emotional and social challenges childless individuals may face, including regret, isolation, discrimination, and stigma—often shaped by pronatalist and heteronormative expectations. Moreover, these interventions should recognise the diverse ways childless individuals perceive and negotiate care, including the types of support they value, the conditions for accepting it, and the networks they rely on. The reviewed literature points to promising practices, such as intergenerational activities, health promotion initiatives that support physical, social, and mental well-being, and accessible support for legal, financial, and end-of-life planning, including support for post-mortem arrangements (Abramowska-Kmon et al. 2023; Silva et al. 2024; Stegen et al. 2021).

This scoping review has several limitations. First, it relies primarily on published studies available in selected academic databases, potentially overlooking certain grey literature such as government reports and policy briefs from NGOs. Second, although all included studies come from academic publications, we did not evaluate their methodological quality—an omission that may impact the reliability of the findings. This decision is consistent with the aims of a scoping review which seeks to broadly map the field rather than critically appraise individual studies. Third, identifying relevant information from the included sources carries a risk of bias. To mitigate this, the authors engaged in iterative discussions throughout the review process, providing an informal layer of peer assessment. Fourth, while consistent search terms and Boolean operators were applied across databases, we acknowledge that adapting these to specific databases might have improved search precision. Lastly, our exclusive focus on original qualitative studies may have limited the inclusion of insights from quantitative and mixed-methods research. Nevertheless, this focus enabled us to capture rich and diverse accounts of the lived experiences of childlessness in midlife and late adulthood. To address these methodological limitations, future reviews should consider adopting a more integrative approach that incorporates quantitative, mixed methods and qualitative studies to provide a more comprehensive understanding of the topic.

## Supplementary Information

Below is the link to the electronic supplementary material.

Supplementary file1 (DOCX 46 KB)

## Data Availability

Data supporting the findings of this scoping review are derived entirely from publicly available sources and articles, which are cited within the manuscript.
